# Interpreting Trends in Self-Efficacy: Why P-Values Alone Do Not Tell the Full Story

**DOI:** 10.31557/APJCP.2026.27.2.403

**Published:** 2026-02-06

**Authors:** Pratap Kumar Jena, Debashish Mohapatra

**Affiliations:** *School of Public Health, Kalinga Institute of Industrial Technology (KIIT) Deemed to be University, Bhubaneswar, India. *

**Dear Editor**

We read with great interest the article “Integrating Dental Professionals for Sustainable Tobacco Cessation: Evaluation of a Capacity Building Intervention” published in APJCP by Aroquiadasse M et al., in the September issue [1]. The study addresses an important and underexplored area, empowering dentists to play an active role in tobacco cessation counseling. We commend the authors for conducting this randomized controlled trial and wish to offer reflections on the interpretation of their findings.

In the abstract, the authors report that no significant differences in knowledge and attitude were observed, but improvement was seen in scores of self-efficacy (Difference of Mean Difference: 2.72, 95% CI: -1.55 – 6.98, p=0.313) and practice behavior (Difference of Mean Difference: 3.49, 95% CI: -1.35 – 8.32, p=0.155) after the intervention. While the phrasing suggests a benefit, both confidence intervals include zero and the reported p-values indicate non-significance. In such intervention research, however, a non-significant p-value does not necessarily negate the importance of the observed effect. Randomized controlled trials with modest sample sizes are often underpowered, and in these contexts, effect sizes, confidence intervals, and practical trends may provide more meaningful insights than p-values alone [2].

Baseline self-efficacy scores for both intervention (39.77±9.71) and control (37.84±7.95) groups were mid-range on the ProSCiTE scale (13–65), likely reflecting the participants’ prior training as dental professionals. This relatively strong starting point may have attenuated the measurable impact of the intervention. Nonetheless, even modest upward shifts in self-efficacy are clinically relevant. Incremental improvements in provider confidence can influence the consistency and quality of cessation counselling, and ultimately support patient quit attempts and long-term abstinence [3, 4].

Another important aspect concerns the treatment of barriers in the study. The ProSCiTE tool evaluates five domains: knowledge, attitude, self-efficacy, practice behavior, and barriers. While the first four were analyzed using the Difference-in-Differences approach, barriers were presented only descriptively. This limits the quantitative interpretation. Interestingly, dentists in the intervention group more often reported “lack of knowledge” as an extreme barrier post-training. Rather than signaling a negative outcome, this may indicate heightened awareness of existing gaps, which is itself valuable. Still, future studies would benefit from analyzing barrier scores systematically, either as continuous or ordinal outcomes, so that all five domains are fully integrated into the intervention assessment.

Overall, this study highlights the promise of engaging dental professionals in tobacco cessation. At the same time, it reminds us that p-values are not the sole lens through which to interpret intervention outcomes. Clinical and educational research often yields incremental shifts that may not reach statistical significance but still carry practical importance. Emphasizing effect sizes, confidence intervals, and contextual interpretation alongside p-values will allow for a more balanced appraisal of capacity-building interventions.

## References

[1] Aroquiadasse M, Lakshminarayanan S, Kar SS, Kubendiran R (2025). Developing a sustainable model for tobacco cessation by integrating dental professionals: An evaluation of a capacity building intervention in pondicherry, india. Asian Pac J Cancer Prev..

[2] Amrhein V, Greenland S, McShane B (2019). Scientists rise up against statistical significance. Nature..

[3] Carson KV, Verbiest ME, Crone MR, Brinn MP, Esterman AJ, Assendelft WJ (2012). Training health professionals in smoking cessation. Cochrane Database Syst Rev..

[4] Sheffer CE, Barone CP, Anders ME (2009). Training health care providers in the treatment of tobacco use and dependence: pre- and post-training results. J Eval Clin Pract..

