# Supplementary material for: Interpreting Trends in Self-Efficacy: Why P-Values Alone Do Not Tell the Full Story
Source: Asian Pac J Cancer Prev. 2026 Feb 6;27(2):403–4. doi: 10.31557/APJCP.2026.27.2.403 (PMC13499981; doi:10.31557/APJCP.2026.27.2.403)
Supplement: Related texts

# *Reply to the letter to the editor: *Interpreting Trends in Self-Efficacy: Why p-values Alone Do Not Tell the Full Story

**Published:** 2026-02-06

**Dear Editor**

We thank the authors for their thoughtful observations and constructive comments on our article.

We appreciate their interpretation that the improvement seen in the domains of self-efficacy and practice behavior, though not statistically significant, does not indicate a lesser effectiveness. We concur that even modest, non-significant positive shifts can have clinical relevance, particularly in early-phase capacity-building interventions.

Regarding the descriptive presentation of barriers, we wish to clarify that this was a deliberate methodological decision. In the pilot phase of our study, we found that tobacco cessation counselling was not generally practiced among dental professionals. Therefore, participants at baseline would not have had adequate experience to meaningfully identify barriers. We therefore chose to document barriers only at the end line for both the intervention and control groups, capturing their reflections after having attempted counselling in practice. This approach enabled us to gather more realistic and experience-based responses.

We acknowledge, however, that including barrier assessments both at baseline (to capture perceived challenges) and at end line (to capture experienced challenges) could provide additional insights in future research. We appreciate the authors’ suggestion in this regard.

We sincerely thank the authors once again for their valuable insights, which reinforce the importance of contextual and practical interpretation in behavioral intervention research.
